# The Role of Arterial Spin Labeling Functional MRI in Assessing Perfusion Impairment of Renal Allografts: A Systematic Review

**DOI:** 10.7759/cureus.25428

**Published:** 2022-05-28

**Authors:** Jayksh Chhabra, Guruprasad Vasant Karwarker, Medha Rajamanuri, Anand Reddy Maligireddy, Eiman Dai, Meher Chahal, Sai Mahitha Mannava, Michael Alfonso

**Affiliations:** 1 Internal Medicine, California Institute of Behavioral Neurosciences & Psychology, Fairfield, USA; 2 Psychiatry, California Institute of Behavioral Neurosciences & Psychology, Fairfield, USA; 3 Pediatrics, California Institute of Behavioral Neurosciences & Psychology, Fairfield, USA; 4 Medicine, California Institute of Behavioral Neurosciences & Psychology, Fairfield, USA

**Keywords:** asl fmri, renal transplantation., chronic renal failure, renal allograft, renal perfusion imaging, renal magnetic resonance

## Abstract

Arterial spin labeling (ASL) is a functional magnetic resonance imaging (fMRI) technique that uses water in arterial blood as a tracer to map an area of interest where the intravascular and extravascular compartments exchange. Our review article focuses primarily on the role of ASL fMRI in assessing perfusion impairment in renal allografts in order to take appropriate steps to eliminate the cause of perfusion impairment at an early stage, thereby extending graft life. The study also highlights various other fMRI techniques that are used to analyze other parameters that affect kidney transplants both acutely and chronically. We gathered our data in accordance with the 2020 Preferred Reporting Items for Systematic Reviews and Meta-Analyses (PRISMA) guidelines, and our search strategy included exclusion/inclusion criteria. Several databases were used in the search strategy, including PubMed, Cochrane, and Science Direct, and the Medical Subject Headings (MeSH) strategy was specifically used for PubMed, and two people scrutinized those papers to conclude that a total of 10 research papers are included in our study. This review article includes papers involving 20 to 98 subjects who had renal allografts within the previous six months and had renal cortical perfusion values measured by ASL fMRI ranging from 35 to 304 ml/100 g/min. Furthermore, when compared to healthy kidney transplant patients, renal ASL perfusion values were significantly lower in subjects with the functional imbalance of kidney transplants. It had a positive correlation with the estimated glomerular filtration rate (eGFR). To summarize, ASL fMRI is critical in detecting renal allograft perfusion impairment.

## Introduction and background

According to the Centers for Disease Control and Prevention's chronic kidney disease (CKD) health policy, more than one in seven, or 15% of US adults, or 37 million people, are estimated to have CKD, and nine out of 10 adults with CKD are unaware of their condition [1]. If CKD is left untreated, it will completely shut down the function and permanently damage the kidneys, eventually leading to end-stage renal disease (ESRD) [2]. While dialysis keeps the patient alive, it only performs 10% of the kidney's function, resulting in dangerous health conditions such as amyloidosis, carpal tunnel syndrome, sepsis, and so on, whereas transplantation is regarded as a long-term treatment because it extends patients' lives with few side effects [2]. Every month, more than 3000 patients are added to the waiting list for kidney transplantation [3]. Dialysis patients have a 15-20% mortality rate after one year of treatment, with a five-year survival rate of less than 50%; on the other hand, transplant recipients have a five-year survival rate of around 80%, making kidney transplantation a better choice [4]. The main issue with kidney transplantation is the scarcity of organs. As a result, some transplant centers are taking steps to alleviate the shortage by using kidneys from older donors or donors who have previously had hepatitis [5].

Kidney transplantation is superior to dialysis in the treatment of ESRD patients because it improves survival rates and quality of life [6]. However, this intervention presents a significant challenge to today's doctors. They must be aware of the numerous negative side effects of immunosuppression, which is used to prevent immune-mediated graft rejection [7]. Ischemia-reperfusion injury (IRI) frequently causes non-specific inflammatory responses during renal transplantation, which may result in kidney graft viability loss [8]. IRI has also been linked to kidney allograft dysfunction and acute rejection, both of which decrease graft survival [9]. Inadequate blood supply to the grafts is also seen as a result of edema and interstitial inflammation, which eventually cause acute graft injury and increase the risk of chronic graft failure [10].

Some studies have mentioned the importance of renal functional magnetic resonance imaging (fMRI) in assessing the function of kidney transplants in light of recent advances in the diagnostic field. Functional MRI techniques include a variety of noninvasive methods for assessing perfusion defects and interstitial fibrosis in grafts. It also avoids the use of potentially harmful agents such as gadolinium and invasive microprobes to detect oxygen levels [11-12]. Imaging techniques include blood oxygenation level-dependent (BOLD) fMRI, diffusion-weighted (DWI) fMRI, dynamic contrast-enhanced (DCE) fMRI, and arterial spin labeling (ASL) fMRI. ASL fMRI has been at the forefront of assessing perfusion in kidney transplants using endogenous tracers [13]. ASL fMRI was first used to measure problems with blood flow in the brain, but as technology improved, it became possible to use ASL fMRI on organs in the abdomen as well, with the kidney being the first organ to use ASL fMRI [14].

In our review, we discovered that the use of ASL fMRI is not widely used in clinical settings, but many studies have addressed the use of ASL fMRI to detect renal rejection in kidney transplant recipients. Furthermore, there was a scarcity of literature indicating its use on a large clinical scale with real cost-effectiveness. As a result, researchers should concentrate on these aspects to improve the use of ASL fMRI in the clinical setting to control graft rejection in patients. We will talk about renal allograft rejection caused by poor blood flow and how ASL fMRI can help find this problem in renal allografts.

## Review

Methods

We completed our review article by taking into account Preferred Reporting Items for Systematic Reviews and Meta-Analysis (PRISMA) guidelines 2020 [15].

Qualifying/Inclusion Criteria

To be added to this paper, the studies have to meet these specific eligibility criteria. All the articles should have been published in the last 10 years and must be in the English language; papers must have renal allografts as their subjects. We included observational studies, cross-sectional studies, and case reports.

Exclusion Criteria

Papers with the animals and pediatric (<18) age group as their subjects were excluded from our study. Also, the information in the grey literature books was not included.

Data Sources and Literature Review

Two reviewers performed a search strategy using various keywords such as renal transplantation, chronic renal failure, renal allograft, renal perfusion imaging, renal magnetic resonance, ASL fMRI, including Medical Subject Headings (Mesh) strategy keywords in PubMed, Science Direct, and Cochrane databases.

We specifically confined our advanced search strategy to PubMed, which yielded 819 results, and other databases such as Science Direct and Cochrane produced 740 and 23 papers, respectively. After completing the search strategy, we imported our records into Endnote 2.0, removing duplicates (n=5). Two investigators screened the papers individually for the accuracy of title/abstracts, full text, and abstracts only, which excluded 1500 records. We delved deeper and explored various references of the articles retrieved to find additional relevant information that might have been missed or overlooked.

Study Selection

**Figure 1:**
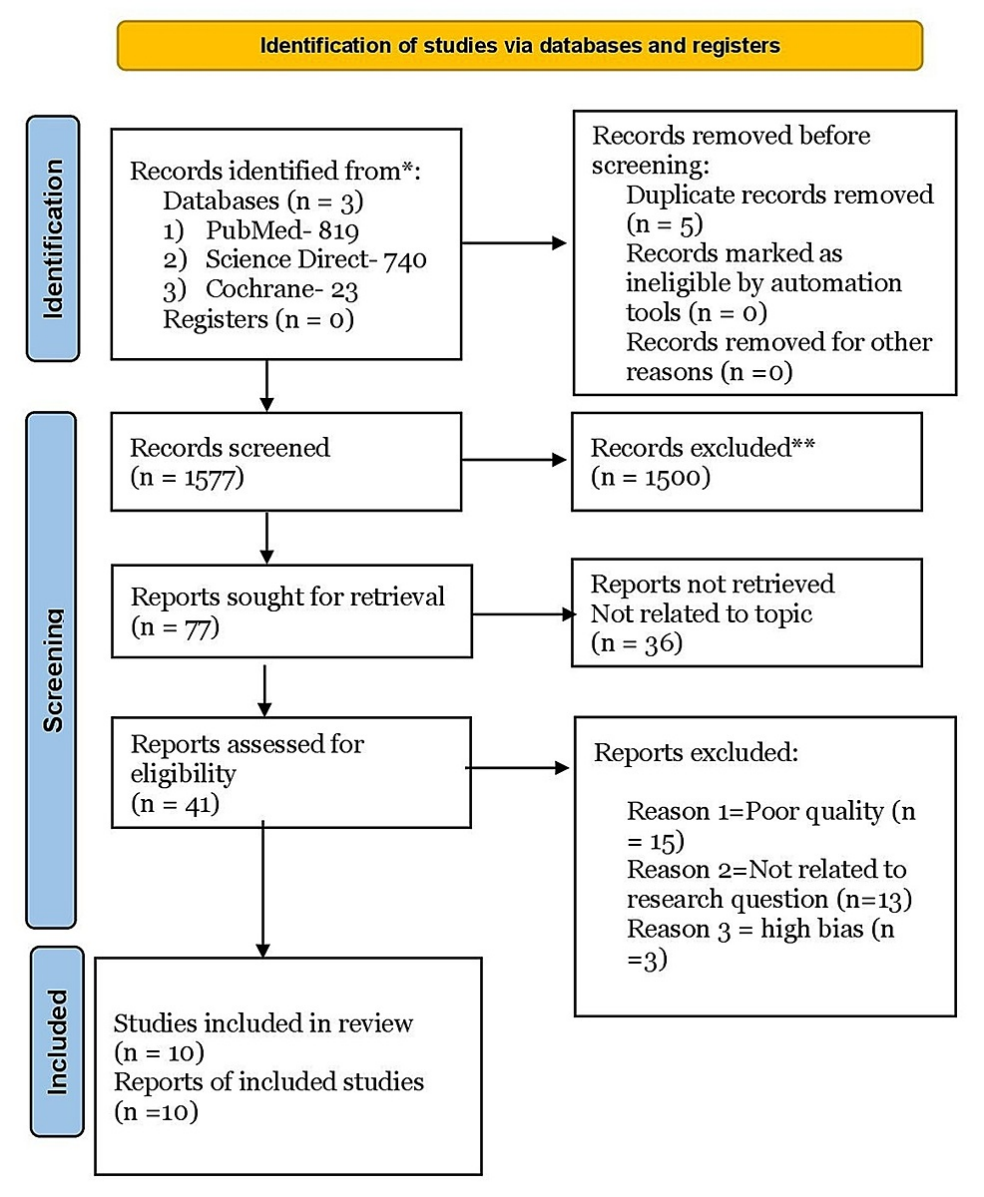
Data extraction process using the PRISMA flowchart 2020 * the number of records identified from each database or register searched (rather than the total number across all databases/registers) ** the number of records were excluded by a human and by automation tools PRISMA - Preferred Reporting Items for Systematic Reviews and Meta-Analysis

All the papers were thoroughly screened and checked for the inclusion criteria, which states that articles should be published in the last 10 years, written in English, and only include kidney transplant patients. The studies which took interstitial fibrosis and perfusion of renal allografts into account were chosen for the results section. A detailed description of the data extraction using the PRISMA flowchart is illustrated in Figure 1 [15].

Quality Assessment

The quality of all the 41 selected records was assessed based on different tools for other types of studies given, and out of those, we excluded 31 papers based on poor quality, such as the Cochrane risk bias assessment tool for a randomized clinical trial, New castle Ottawa scale for observational studies, CARE guidelines (CAse REports) for case reports and PRISMA guidelines 2020 for systematic reviews and meta-analysis.

Results

After the screening process, 10 articles that were properly reviewed and included in the results section were carefully looked at by two investigators working together. The results of these 10 studies were then extracted on a separate spreadsheet made in Microsoft Excel 2019 to make the data extraction form.

The studies chosen for inclusion in the final review primarily addressed the underlying cause of the transplant's functional abnormality, assessing the function of the grafts using various fMRI techniques that are non-invasive and more beneficial than other diagnostic tools. We focused on 10 studies that used ASL fMRI to find perfusion problems in the cortical area of grafts and link them to estimated glomerular filtration rate (eGFR) values, which are a measure of how well the kidneys are working.

The studies included 20-98 patients, all of whom had renal allografts with or without clinical problems. Only one of the four articles took the reading after 24 hours to check the test's intra-visit reproducibility. In many transplant patients, ASL fMRI determined renal cortical perfusion ranging from 35 to 304 mL/100 g/min. A few papers examined the physiological abnormalities that were used to perform the renal ASL fMRI. When compared to healthy kidney transplant patients, values of renal ASL perfusion were much lower in subjects with a functional imbalance from kidney transplants.

The discussion section included five research papers that used various fMRI techniques to analyze other issues such as interstitial fibrosis and blood oxygen levels in renal grafts. The patients ranged from 21 to 122 and included both DWI and BOLD fMRI subjects. The DWI fMRI discussed here is used to assess the amount of fibrosis in the grafts, which is thought to be the most important cause of chronic renal allograft dysfunction. In BOLD fMRI, the R2 (transverse relaxation rate expressed as per second) value is used as a single parameter to figure out how much oxygen is in a graft patient's blood and how fast their bodies work.

Discussion

In terms of survival, quality of life, and cost-effectiveness, patients with end-stage renal disease have a better prognosis with kidney transplantation than with dialysis [16]. Although Doppler ultrasound, urinalysis, and renal biopsy are thought to be the most reliable methods of detecting renal allograft dysfunction, recent technological advancements have revealed that functional MRI can provide significant non-invasive and non-nephrotoxic results [17]. ASL fMRI is a type of functional MRI that can be used to find out if there is a problem with blood flow, which can lead to a decrease in plasma blood flow (PBF), eGFR, and eventually allograft dysfunction [18].

Renal Functional MRI

**Figure 2:**
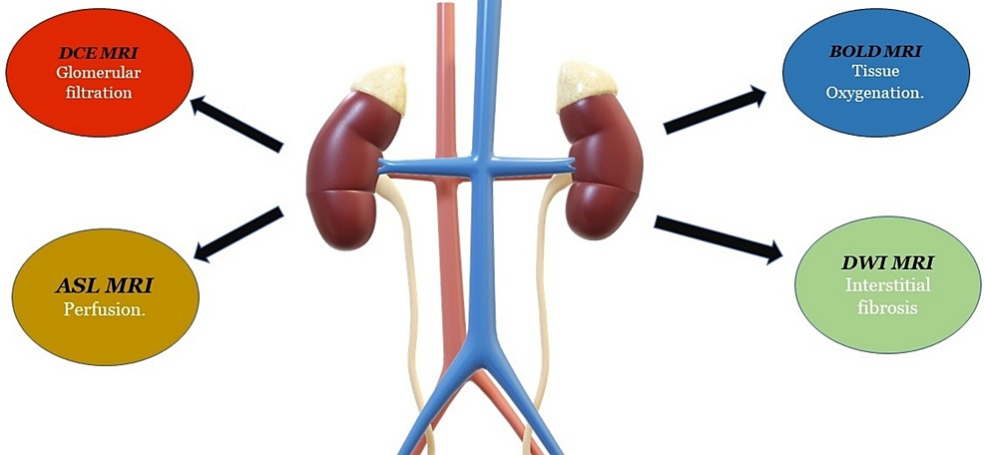
Various fMRI techniques with their functions fMRI - functional magnetic resonance imaging; BOLD fMRI - blood oxygen level-dependent fMRI; DWI fMRI - diffusion-weighted fMRI; DCE fMRI - dynamic contrast-enhanced fMRI; ASL fMRI: arterial spin labeling fMRI The original image is owned by the corresponding author.

As problems can arise in both the early and late stages of renal allograft dysfunction, it is critical to detect them as soon as possible [19]. Preventing early loss of transplant functions and beginning treatment of the allograft's underlying dysfunction has been made possible through the use of renal functional MRI in screening kidney allografts for interstitial fibrosis, perfusion impairment, and tissue oxygenation [20]. Many fMRI techniques have been used to evaluate the specific functions of the foreign kidneys for the parameters listed above. It is possible to check tissue oxygenation and cortical perfusion using BOLD fMRI and ASL fMRI, respectively, while eGFR can be assessed with DCE fMRI, and interstitial fibrosis can be detected using DWI fMRI [21]. Figure 2 depicts a variety of fMRI techniques used to analyze renal grafts.

According to the literature, much research has been done on various renal functional fMRI techniques briefed in the above figure. A considerable alteration in the cortical and medullary perfusion can cause moderate renal failure, as demonstrated by Rossi et al. study [22].

Arterial Spin Labeling (ASL) Magnetic Resonance Imaging

ASL is an fMRI technique that magnetically labels the water in arterial blood and uses it as a tracer to map an area of interest where the intravascular and extravascular compartments exchange. Furthermore, no nephrotoxic agents such as gadolinium are required [23]. Renal artery stenosis is one of the complications in kidney transplants that can cause an imbalance in the kidney blood supply and, eventually, function loss because perfusion is the primary driving force for the eGFR to excrete waste products [23]. As a result, if there is a perfusion impairment in kidney transplants, patients can be screened for and treated for renal artery stenosis.

The label used in the ASL fMRI, according to Wong et al., is an arterial blood label, which means it is less likely to become mixed up with the venous circulation. Furthermore, it produces an fMRI result on the 10th of a second and is retained in the tissue to which it was administered [24]. Moreover, when compared to biopsy and other diagnostic methods, ASL fMRI has several advantages, such as it does not use ionizing radiation or nephrotoxic contrast, which reduces the risk of contrast-related kidney damage [25].

ASL fMRI: A Correlation Between Renal Function and eGFR

Heusch et al. conducted a prospective study on non-contrast-enhanced ASL fMRI on renal allograft patients [26]. Their research chose two magnetic fields that are 1.5T and 3T, with 98 patients and divided them into two groups, group A with adequate functioning grafts with eGFR>30 mL/min/1.73 m^2^ and group B with eGFR <30 mL/min/1.73 m^2^. After comparing the results at 1.5T and 3T, they seemed to be comparable, and we interpreted that the group with low eGFR tends to have decreased renal function and the value of ASL perfusion was also down in this group. Therefore, group B proved a strong correlation between ASL, renal function, and eGFR values. 

Artz et al. worked on a prospective study in which they chose 25 subjects, including some native and transplanted kidney patients [27]. They used only a 1.5T magnetic field and kept eGFR=60 mL/min/1.73 m^2^ as the threshold value and interpreted the results by looking above and below this value. According to their study, there is a correlation between eGFR and cortical perfusion. In the group with eGFR>60 mL/min/1.73 m^2^, the cortical perfusion was more native than transplanted. But, on the contrary, in the group with eGFR<60 mL/min/1.73 m^2^, medullary perfusion was increased compared to the transplanted group. The chances of bias are higher in Artz et al. than in Heusch et al., given that fewer patients were in this study [26-27]. 

ASL fMRI: Cortical vs. Medullary Perfusion

In their study, Artz et al. took 24 patients, out of which 14 were transplanted patients and 10 were native patients [28]. They used unique ASL techniques known as flow-sensitive alternating inversion recovery (FAIR) ASL, and they recorded the results 24 hours apart to check the inter visit perfusion impairment in subjects. According to their findings, ASL is a trustworthy method for assessing renal function in the cortex of native and transplanted kidney patients since it recreated a wide range of renal activities. On the flip side, there was very little documentation of medullary perfusion in the subjects after 24 hours; overall, it proves that ASL fMRI, especially FAIR ASL fMRI, is quite effective in analyzing the cortical perfusion.

Lanzman et al. worked on 20 subjects and divided them into three groups [29]. One group consisted of the subjects with stable allograft function, the other group with good functioning and underwent transplantation in the previous three weeks. The last group consisted of the issues with functional deterioration. They performed ASL fMRI and observed that ASL perfusion values were least in the previous group with functional impairment, proving that ASL fMRI is an excellent technique for detecting cortical perfusion compared to medullary perfusion. This study has fewer patients than Artz et al. [28]. Therefore, the chances of bias are higher in this study.

Moreover, this study also mentions the lack of fine shreds of evidence to detect the clinical value of ASL to monitor and screen renal allograft functional loss. Also, we have proposed a systematic approach to see any perfusion impairment in the grafts. A checklist should be used so that no other important step goes unnoticed, which ultimately affects the result of the experiments. A representation of such an index has been given in Table 1 [23].

**Table 1:** Checklist to report ASL studies ASL - arterial spin labeling; BOLD - blood oxygenation level-dependent; DWI - diffusion-weighted Adapted from Odudu et al. [23].

Checklist for reporting ASL studies
The patient should be well prepared with adequate water intake, and well-detailed history should be taken.
Document vitals such as blood pressure, pulse rate, and temperature.
Intake of all the medications especially targeting the renin aldosterone system.
Brief the patient about all the details of the experiment.
A T1 map can be used to assess the progress of the experiment.
Document the duration of delay after labeling.
If possible, focus on other MRI parameters also like tissue oxygenation, fibrosis (BOLD, DWI).
Medullary and cortical perfusion values should be mentioned differently.

ASL fMRI and Intravoxel Incoherent Motion (IVIM)

Ren et al. used intravoxel incoherent motion (IVIM) and ASL fMRI on renal allografts to evaluate their function early after transplantation [30]. A total of 82 participants, including 62 renal allograft recipients (two-four weeks after kidney transplantation) and 20 volunteers, were enrolled to be scanned on a 3.0T MR scanner using IVIM and ASL fMRI. Recipients were separated into two groups based on their estimated glomerular filtration rate (eGFR) with a threshold of 60ml/min/1.73m: normal or impaired function. The apparent diffusion coefficient (ADC) of pure diffusion (ADCslow), the ADC of pseudo diffusion (ADCfast), the perfusion fraction (PF), and the renal blood flow (RBF) of the cortex were compared between three groups. The relationship between ADCslow, ADCfast, PF, RBF, and eGFR was investigated [30].

There was no significant difference in mean cortical ADCslow, ADCfast, and PF in allografts with normal function than healthy controls (p>0.05). The mean cortical ADCslow, ADCfast, PF, and RBF for allografts with impaired function were lower than those with normal function (p<0.05). For recipients, mean cortical ADCslow, ADCfast, PF, and RBF positively correlated with eGFR (all p=0.01). Therefore, it can be interpreted that cumulative IVIM and ASL fMRI can assess allografts' diffusion and perfusion properties [30].

Various fMRI Techniques

Other non-invasive fMRI techniques such as DWI fMRI and BOLD fMRI focus mainly on detecting the interstitial fibrosis and oxygen level in renal allografts, respectively.

DWI fMRI and Interstitial Fibrosis

Beck-Tölly et al.'s research revolved around the interstitial fibrosis (IF) of the allografts, considered the primary cause of chronic allograft injury in kidney transplants [31]. The quantity of IF was measured using cortical T1 relaxation time, which was directly proportional to the degree of fibrosis in the tissue. As part of their investigation, Hueper et al. coupled diffusion tensor imaging (DTI) and DWI fMRI to detect an early functional decline in kidney transplants [32]. It was shown in another study that the fMRI parameters and the amount of fibrosis could be linked at an early stage to detect graft damage and prevent it at an early stage [32].

BOLD fMRI and Oxygenation

BOLD fMRI uses the R2 value, a measure of deoxyhemoglobin in the kidney, and its decrease indicates higher free oxygen availability in the kidney and vice versa. Sławińska et al. experimented on patients with early kidney transplantation to detect the blood oxygenation level in the allografts, and they noticed a minimal change in the R2 value, which is a parameter of BOLD fMRI [33]. Hence it didn't provide any predictive value for the future. On the other hand, Sadowski et al. conducted a clinical trial using BOLD fMRI and contrast-enhanced perfusion fMRI [34]. They concluded that compared to patients with acute tubular necrosis and normal allograft function, oxygen availability in acute rejection patients was increased while medullary perfusion was significantly reduced. Xiao et al. also experimented on patients with acute renal rejected kidney transplants, and they interpreted that decrease in cortex and medulla R2 value and the R2 ratio of medulla/cortex (M/C) suggest that patient has an acute rejection of the transplant [35]. Table 2 summarises all the information about the author, type of study, and their results.

**Table 2:** Author names, type of studies, and results interpreted from different studies MRI - magnetic resonance imaging; BOLD MRI - blood oxygen level-dependent MRI; DWI MRI - diffusion-weighted MRI; DCE MRI - dynamic contrast-enhanced MRI; ASL MRI - arterial spin labeling MRI; IVIM - intravoxel incoherent motion; ADCslow - apparent diffusion coefficient of pure diffusion; ADCfast - apparent diffusion coefficient of pseudo diffusion; PF - perfusion fraction; RBF - renal blood flow; FAIR - flow-sensitive alternating inversion recovery; IF - interstitial fibrosis

Author/year	Type of study	Subjects	Characteristics	Results
Heusch et al. 2014 [26]	Prospective cohort	Total=98 allograft recipients. 1) group A >30 mL/min/1.73 m^2^, 2) Group B <30 mL/min/1.73 m^2^	Renal perfusion and GFR correlation	ASL renal perfusion values were low in group B, whose GFR values were already low and marked as deteriorated renal function. Therefore, it proved that ASL has a link with eGFR.
Artz et al. 2011 [27]	Prospective case-control	Total=25 subjects. 1) native=10, 2) transplant=15	Renal perfusion and GFR correlation	Native kidney patients with eGFR >60 mL/min/1.73 m^2 ^showed higher ASL cortical perfusion value and with eGFR <60 mL/min/1.73 m^2 ^showed medullary perfusion. In transplanted patients with eGFR >60 mL/min/1.73 m^2, ^there was sufficient cortical perfusion compared to the allograft group with eGFR< 60 mL/min/1.73 m^2.^.
Artz et al. 2011 [28]	Prospective case-control	Total=24 subjects. 1) native kidney=10, 2) transplant kidney=14	Reproducibility of ASL renal perfusion technique in cortical vs. medullary perfusion	Results interpreted that the FAIR ASL technique produces more accurate results in the cortex region than in the medullary region.
Lanzman et al. 2010 [29]	Prospective cohort	Total=20 patients. 1) group A - six patients with stable allograft over four months, 2) group B - seven patients with good allograft function who underwent transplants in the previous three weeks, 3) group C - seven recipients with an acute decrease in renal function	Perfusion evaluation in various groups of allograft patients	ASL proved to be an excellent technique to detect perfusion impairment in transplants as there was a significant decrease in cortical perfusion in group c, as verified by ASL.
Ren et al. 2016 [30]	Prospective cohort	Total=82 subjects. 1) renal allograft recipients=62, 2) volunteers=20	IVIM and ASL MRI to assess the diffusion and perfusion properties of allografts	1) With normal functions of allografts: no significant difference in mean cortical ADCslow, ADCfast, and PF in allografts with normal function compared to healthy controls. 2) With impaired function allografts: the mean cortical ADCslow, ADCfast, PF, and RBF for allografts with poor function were lower than those with normal function. 3) For recipients: mean cortical ADCslow, ADCfast, PF, and RBF all showed a positive correlation with eGFR.
Beck-Tölly et al. 2020 [31]	Prospective center trial	Total=32 patients	Correlation between interstitial fibrosis and T1 relaxation time	The value of cortical T1 MRI is directly proportional to the amount of IF present in the graft. It is supported by the presence of protein in the urine, considered a marker for detecting the long-term prognosis of the allografts.
Heuper et al. 2016 [32]	Case-control	Two groups: 1) 33 people with normal grafts, 2) 31 patients with abnormal graft function	Combined DWI and DTI for detection of allograft dysfunction	DWI and DTI demonstrated reduced diffusion anisotropy or diffusivity restriction in patients with delayed graft function.
Sławińska et al. 2018 [33]	Case-control	Total=50 subjects. 1) case=40 (one week after kidney transplantation), 2) control=10	Correlation of R2 value and blood oxygenation levels in the allografts using BOLD MRI	Minimal change in R2 value was detected in the subjects. Therefore it could not see the exact importance of the R2 value in detecting the oxygen level in grafts.
Sadowski et al. 2010 [34]	Clinical trial	Total=21 subjects. 1) male=13, 2) female=8	Comparison of R2 values of patients with acute rejection, acute tubular necrosis (ATN), and normal renal function using BOLD MRI and contrast-enhanced perfusion MRI	Oxygen bioavailability increased, and R2 value decreased in the acute renal rejection patients compared to the patients with ATN and normal renal function.
Xiao et al. 2012 [35]	Case-control	Total=122 subjects. 1) volunteer=20, 2) patients with functional kidney transplants=72, 3) patients with acute rejection=21, 4) patients with acute rejection on follow-up=9	BOLD MRI to assess the oxygen metabolism in standard functioning kidney transplants to distinguish between normal and rejected grafts	Decreased cortex and medulla R2 value and ratio of R2 in medulla/cortex (M/C) suggest that the patient has an acute rejection of the transplant. It helps in distinguishing between normal functioning kidneys and transplant rejected kidneys.

Limitations

**Figure 3:**
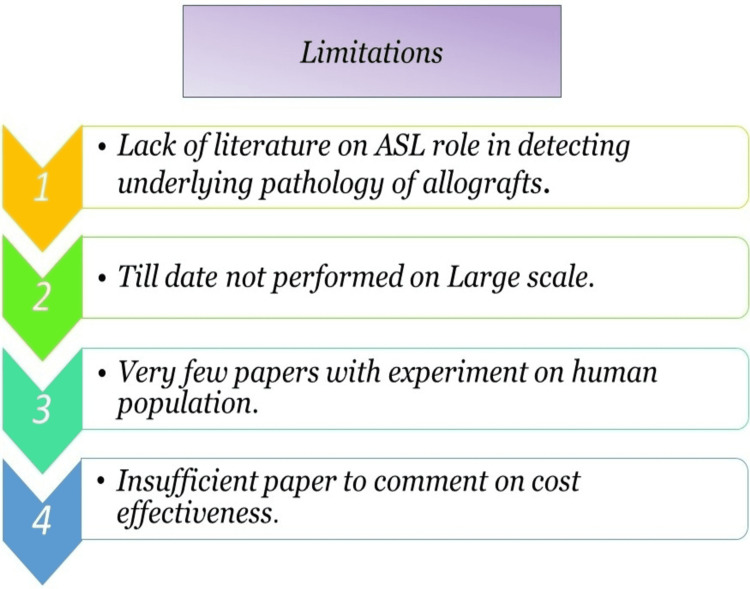
Limitations faced at the time of review ASL - arterial spin labeling The original image is owned by the corresponding author.

While reviewing this, we discovered some limitations that make imposing the ASL fMRI in the clinical setting difficult, such as a lack of data on the generalization of the use of ASL fMRI, a lack of research with a significant number of patients in the experiment, making it difficult to implement in clinical settings. Furthermore, there is no gold standard against which to compare the results of ASL perfusion values. More studies on animals were conducted than on human subjects, making it difficult to apply to the general population. Because there were few papers focusing on the cost of the ASL fMRI, it is impossible to say whether it is cost-effective or not to be applied to the general population. Figure 3 depicts the limitations of our review article.

## Conclusions

After reviewing all of the studies included in this review, it is possible to conclude that ASL fMRI has played an important role in detecting cortical perfusion impairment in renal allograft patients and has demonstrated an important link with eGFR values, which are thought to be a marker of renal function. ASL fMRI should be performed within the first six months to detect any decline in eGFR values, allowing renal impairment in renal allografts to be detected early. Although multiple functional MRI techniques, such as BOLD fMRI, DWI fMRI, and ASL fMRI, are available to detect early loss of function, only the latter has been approved to detect any perfusion-related pathology in the grafts, which is the leading cause of acute graft rejection. It's also worth noting that future research should look for specific ASL fMRI parameters to monitor the renal allograft for rejection. Furthermore, it is suggested that future researchers focus more on ASL fMRI in a large scale population to reduce the possibility of bias in the results.
